# Unobtrusive cot side sleep stage classification in preterm infants using ultra-wideband radar

**DOI:** 10.3389/frsle.2023.1150962

**Published:** 2023-06-15

**Authors:** Emad Arasteh, Eline R. de Groot, Demi van den Ende, Thomas Alderliesten, Xi Long, Robbin de Goederen, Manon Benders, Jeroen Dudink

**Affiliations:** ^1^Department of Neonatology, University Medical Center Utrecht, Wilhelmina Children's Hospital, Utrecht, Netherlands; ^2^Department of Electrical Engineering (ESAT), STADIUS Center for Dynamical Systems, Signal Processing and Data Analytics, KU Leuven, Leuven, Belgium; ^3^Department of Electrical Engineering, Eindhoven University of Technology, Eindhoven, Netherlands; ^4^Brain Center Rudolf Magnus, University Medical Center Utrecht, Utrecht, Netherlands

**Keywords:** sleep stages, radar, respiration, preterm infants, NICU

## Abstract

**Background:**

Sleep is an important driver of development in infants born preterm. However, continuous unobtrusive sleep monitoring of infants in the neonatal intensive care unit (NICU) is challenging.

**Objective:**

To assess the feasibility of ultra-wideband (UWB) radar for sleep stage classification in preterm infants admitted to the NICU.

**Methods:**

Active and quiet sleep were visually assessed using video recordings in 10 preterm infants (recorded between 29 and 34 weeks of postmenstrual age) admitted to the NICU. UWB radar recorded all infant's motions during the video recordings. From the baseband data measured with the UWB radar, a total of 48 features were calculated. All features were related to body and breathing movements. Six machine learning classifiers were compared regarding their ability to reliably classify active and quiet sleep using these raw signals.

**Results:**

The adaptive boosting (AdaBoost) classifier achieved the highest balanced accuracy (81%) over a 10-fold cross-validation, with an area under the curve of receiver operating characteristics (AUC-ROC) of 0.82.

**Conclusions:**

The UWB radar data, using the AdaBoost classifier, is a promising method for non-obtrusive sleep stage assessment in very preterm infants admitted to the NICU.

## Introduction

Very preterm infants (e.g., infants born <32 weeks of gestation) spend the first weeks of their life developing in an incubator rather than within the protective environment of the mother's womb. These infants are particularly vulnerable, as the late second and third trimesters of pregnancy are critical periods for fetal brain development during which new connections are formed (Kostovi et al., 2019). In the womb, fetal sleep is believed to be the major driver of neural activity, a process that is essential for neuronal survival, axonal guidance, and synapse maturation (Vanhatalo et al., 2005; Rio-Bermudez et al., 2020). However, in the neonatal intensive care unit (NICU), preterm infants are exposed to a myriad of extrinsic stimuli that can radically alter their sleep-wake states (Peirano and Algarín, 2007; Graven and Browne, 2008; Besedovsky et al., 2012; Tham et al., 2017). Continuous monitoring of neonatal sleep in the NICU might support care in two ways. First, because of the important role of neonatal sleep in development, it may serve as a biomarker for future outcomes, such as the maturational state and illness severity of an infant (Scher and Loparo, 2009). Secondly, if sleep is monitored in real-time, elective care can be adapted to sleep stages, ensuring optimal (brain) development (Graven and Browne, 2008; Colombo et al., 2011; Allen, 2012).

The main gold standard of sleep assessment in very preterm infants is currently behavioral observation (de Groot et al., 2022). Sleep consists of three behavioral stages. Active sleep (AS) is thought to be important for developing new connections in the brain. Quiet sleep (QS) is thought to be essential for consolidating connections and recovery, and intermediate sleep is thought to be a transitory stage (Knoop et al., 2021). Ideally, elective care is adjusted to neonatal sleep stages. However, to achieve this, continuous sleep observations are required; this is time-consuming and expensive. In behavioral sleep stage classification, heart rate and respiratory rate are important features in addition to visual observation of the behavior of the infant (de Groot et al., 2021). Contact-based sleep monitoring methods like polysomnography (PSG) are difficult to utilize for infants specially inside medical environments (Agnew et al., 1966; Tamaki et al., 2005). As a result, there is a trend to monitor sleep stages in these vulnerable patients through contactless methods (de Goederen et al., 2021). Algorithms based solely on cardiorespiratory parameters seem only moderately reliable (Werth et al., 2019; Sentner et al., 2022). As a result, there is a demand for new or complementary methods and algorithms that can continuously keep track of sleep stages with a higher rate of accuracy.

Several technologies have been proposed for non-contact monitoring of movement and vital signs in the neonatal population (Werth et al., 2017). A promising method is ultra-wideband (UWB) radar (de Goederen et al., 2021). He et al. (2020) have shown that–for five adult subjects–a high-resolution contactless UWB radar can monitor respiration rate more accurately than contact-based methods in the presence of considerable movement artifact. Furthermore, UWB radar is a pulse-based radar that can reliably and non-obtrusively capture vital signs in humans (Walid et al., 2021); it can measure both breathing rate and movement. An UWB radar can discriminate between different objects with a high rate of specificity (Barrett, 2001). It can detect human presence and movement up to 10 m with high spatial resolution using signals that are able to pass through almost any non-organic material (e.g., plastic, blankets and metals) (Walid et al., 2021). Finally, the UWB radar can be used in the dark, creating an advantage over using video-based algorithms, as preterm infants spend most of their time in relatively dark environments [this is already proven to be important in the development of some physiological mechanisms like circadian systems among preterm infants (Hazelhoff et al., 2021)].

UWB radar has already been used successfully to classify sleep stages in older children (de Goederen et al., 2021). However, the use of UWB radar has been investigated only once in a series of 4 infants, including one preterm infant, to distinguish between sleep and wake states (Lee et al., 2021).

In summary, there is a gap in literature about automated sleep classification of preterm infants in a medical environment. The majority of previous studies use term born infants or children (de Goederen et al., 2021), techniques that do not include movement-features (Werth et al., 2017, 2019), techniques that require additional electrodes (Ansari et al., 2018) or they use assessment methods that require high computing power (Cabon et al., 2019). Furthermore, varying–albeit low–performance of these existing algorithms shows that a well-rounded state of the art technique is not available yet. Finally, the existing UWB radar-based techniques have not yet explored additional features besides amplitude (Lee et al., 2021).

As the separate sleep stages (active sleep and quiet sleep) provide important information about sleep quality, the aim of the current study is to assess the feasibility of using UWB radar to continuously measure separate sleep stages in preterm infants admitted to a NICU. Preterm infants show more movement and more irregular breathing patterns in AS compared to QS (de Goederen et al., 2021; de Groot et al., 2022). Therefore, to improve algorithm performance in sleep stage classification, we used movement and respiration (extracted from radar signals) as main features for sleep stage classification. In addition, we utilized the phase of UWB radar data to enhance the classification performance. Furthermore, we integrated empirical mode decomposition (EMD) of the raw radar data into our feature extraction process to capture the non-stationary characteristics and content of sleep-related breathing in very preterm infants.

## Methods

### Study population

All the recordings and evaluations afterward (sleep stages observations) were conducted at the Wilhelmina Children's Hospital in Utrecht, the Netherlands. For all infants, recordings took place between 29 + 0 and 33 + 6 weeks postmenstrual age (PMA). Data was collected from February to October 2022. Exclusion criteria were congenital malformations, a history of seizures, overt brain injury [e.g., intraventricular hemorrhage > grade 2 according to Papile (Papile et al., 1978)], congenital abnormalities and mother's use of recreational drugs during pregnancy. Written informed consent was obtained from parents before enrollment in the study. Permission to use pseudonymized patient data was obtained from parents using the consent form and from the local Medical Ethical Review Committee (METC number 21-816/C). Table 1 lists general patients' characteristics and specific characteristics during radar and video.

**Table 1:** Patient characteristics.

**Sample size**	***n***	**10**
GA at birth	*median (range)*	29 + 6 (25 + 1–31 + 2)
Assigned sex at birth	*%male*	70%
Birth weight	*mean (SD)*	1,253 grams (386)
Apgar score	*median 1/5/10 min*	7/9/8
PMA at observation	*median (range)*	31 + 2 (29 + 0–33 + 4)
**Sleeping position**		
Lateral	*n (%)*	7 (70%) *(5 right/2 left)*
Prone	*n (%)*	1 (10%) *(head right)*
Supine	*n (%)*	2 (20%)
**Mode of ventilation**		
nCPAP	*n (%)*	2 (20%)
nIPPV	*n (%)*	3 (30%)
Optiflow	*n (%)*	2 (20%)
None	*n (%)*	3 (30%)
Medication 24 h before observation + during observation	*n (%)*	Caffeine = 10 Doxapram = 2 Hydrocortison = 1

### Study set-up

**Figure 1:**
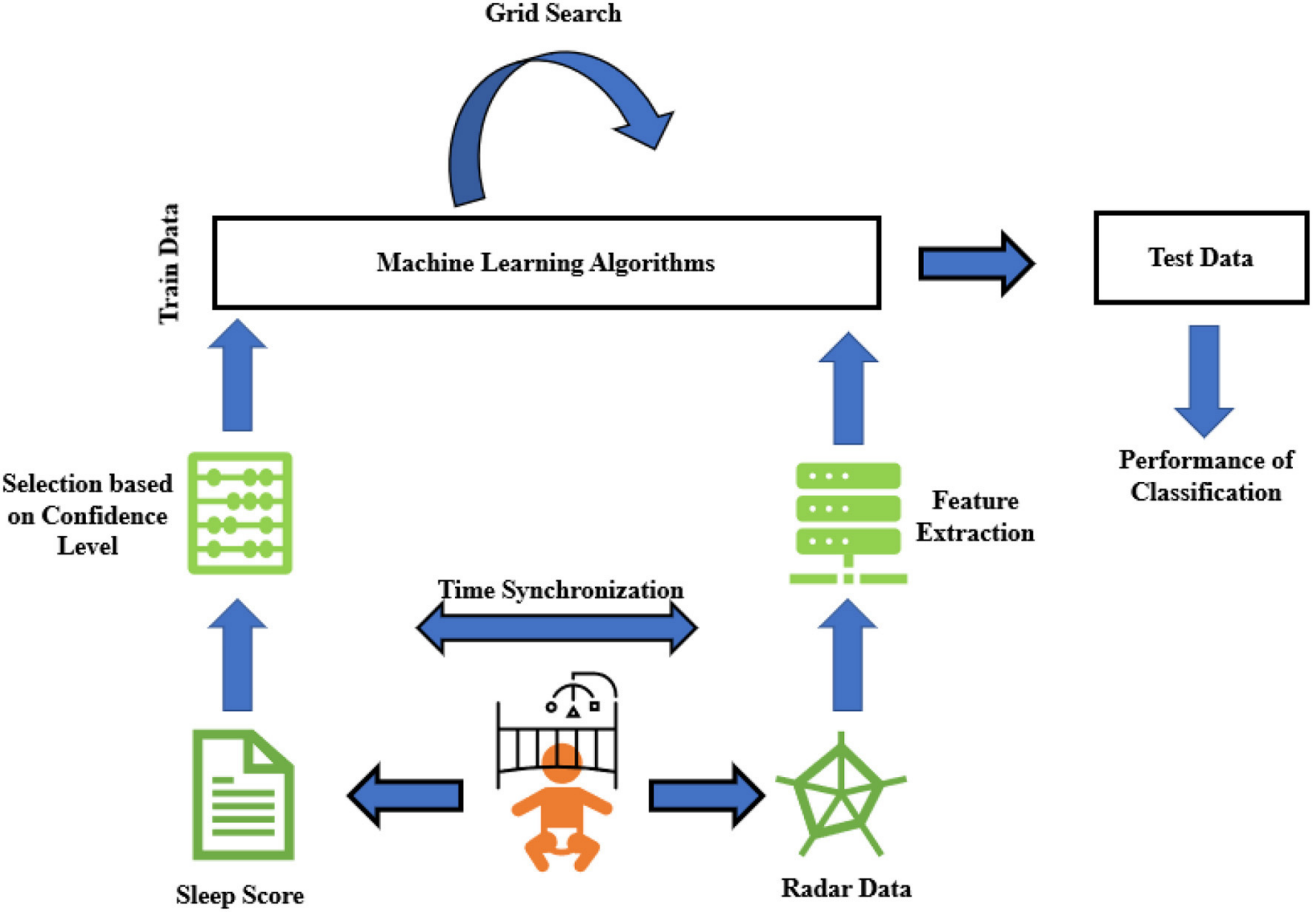
Schematic general overview of sleep stage classification steps in this research. It starts with recording data and scoring sleep observation scores, then continues with feature extraction from those down-converted data. Finally, binary classification is applied to see how accurately we can estimate sleep stages from contactless radar data.

**Figure 2:**
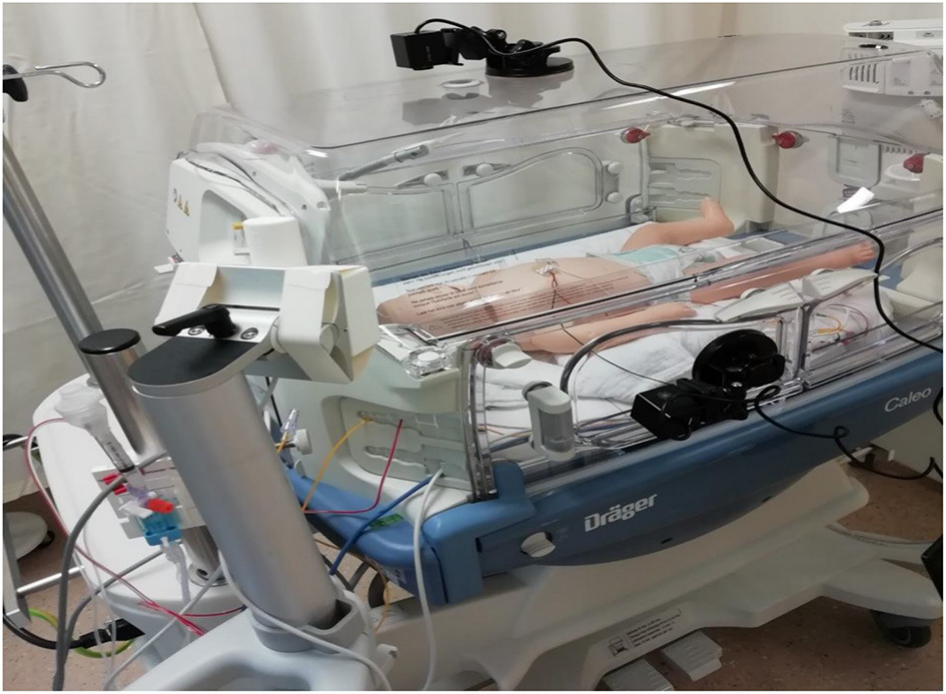
Set up of radar while transceiving pulse waves toward and from preterm infant at neonatology section, children and women hospital, University Medical Center Utrecht, Netherlands.

An overview of the study setup is displayed in Figure 1. Videos were recorded with an Intel^®^ RealSense Depth Camera D435i camera (Intel Corporation, Santa Clara, USA) or a 1SEE VDO360 camera (VDO360, Maryland, USA). Both cameras used 30 frames per second with a resolution of 1,920 × 1,080 pixels. Furthermore, the Xethru X2M200 radar module (Novelda AS, Oslo, Norway) was used (technical specifications are explained in Section Radar data acquisition). Both the video camera and the UWB radar were connected to a standalone laptop next to the incubator. The UWB radar was placed as far away from the regular walkway as possible so as to not be disturbed by signals from other people in the NICU. An overview of the experimental setup is displayed in Figure 2.

### Sleep data acquisition

Sleep observations according to the behavioral sleep stage classification for preterm infants (BeSSPI) sleep classification system (de Groot et al., 2022) were used as a gold standard. All videos were classified by one researcher. Three sleep-wake stages (AS, QS, or W) were assessed in 1-min periods. This study purposely left out IS, which is a transitory stage. Finally, the behavioral observers used the following scales to give a confidence score to every period that indicated how certain they were in their classifications:

- 1 = Highly confident that this sleep stage is correct (80–100% confidence).
- 0 = Relatively confident that this sleep stage is correct (50–80% confidence).
−1 = Not confident that this sleep stage is correct (0–50% confidence).

No smoothing of the data was applied afterward.

### Radar data acquisition

The X2M200 radar module can detect ranges from 0.5 to 2.5 m in 52 bins with sensitivity ranging from 0 to 9 (Bhagat and Raj, 2021). With a higher sensitivity, smaller objects can be distinguished at the cost of more probable false alarms. The radar pulses can be transmitted in two frequency bands of 6.0–8.5 and 7.25–10.2 GHz. For each bin, baseband data is generated 20 times per second. The radar antenna has a 7 dB gain with more than 14 dB front-to-back ratio. The current study utilized 52 bins with a bin length of 0.0388 m. Furthermore, a carrier frequency of 7.46 GHz, a detection zone of 0.4–1 m, and a range offset of 0.3 m were used. Subjects were recorded with a sensitivity of either 5 or 9.

### Radar data processing

**Figure 3:**
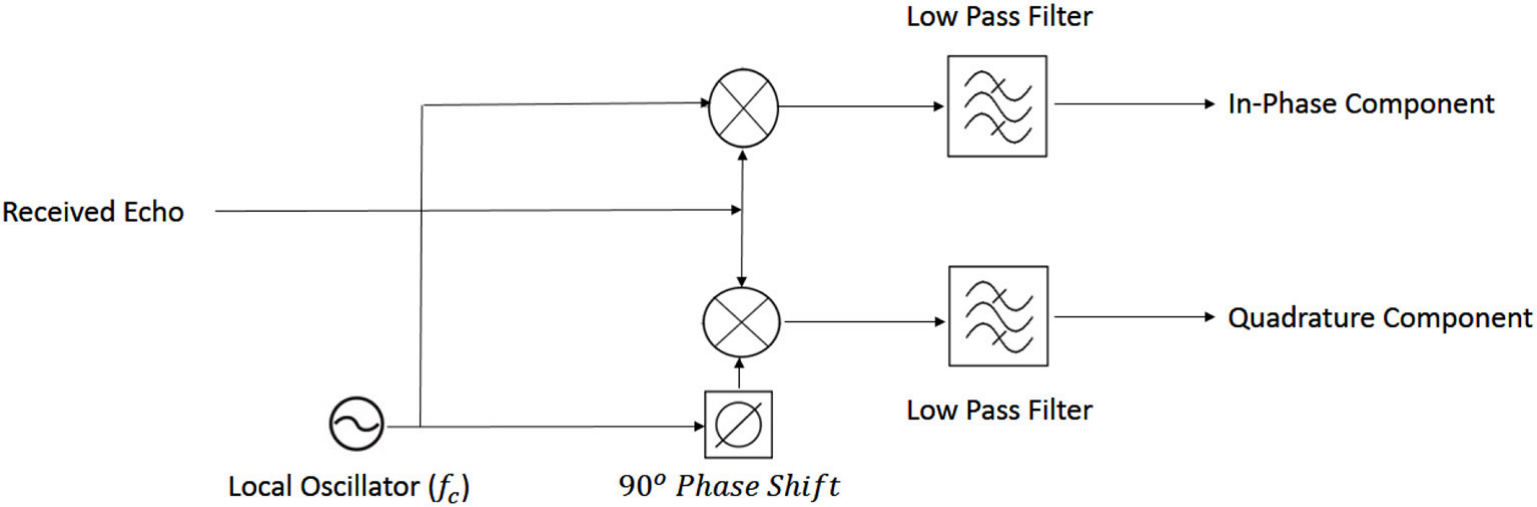
General schematic of down-conversion of radar signal.

The transmitted radio wave is usually called “reference” and the received signal is called “echo” (Skolnik, 1980). The way in which the reference signal is transformed to the echo signal provides the information necessary to detect a target and its specific characteristics. In the current study, the received (echo) signal was decomposed into in-phase and quadrature components (see Figure 3 for a general overview).

Signals are transmitted using a carrier frequency higher than the Doppler frequency content of the target. If the carrier frequency is f_c_, then the received echo signal—influenced by the Doppler frequency of the moving target (*f*_*d*_)—can be written as a signal with lower amplitude *A*′ and a phase shift ∅ [dependent on both the distance between the radar and the target and also on the carrier frequency (Skolnik, 1980)]:


(1) $$\begin{array}{l} S_{echo}=A^{{\;}^{\prime }}\;Sin\left(2\pi \left(f_{c}\pm f_{d}\right)t-\emptyset \right) \end{array}$$

In this formula, the ± symbol is used to distinguish between the approaching or receding target.

After down-conversion (removing the influence of the carrier signal), the baseband can be extracted. The baseband is the frequency band containing the message information about the signal—in our case, the Doppler frequency and speed. To further analyze the distance and speed of the detected object, any carrier frequency leakage in the receiver should be detected and omitted.

Finally, the baseband signal is decomposed into in-phase and quadrature components (Eaves and Reedy, 2012):


(2) $$\begin{array}{l} \text{I}(\text{in}-\text{phase})=\text{LPF}\left\{S_{echo}^{*}cos\left(2\pi f_{c}t\right)\right\}\\ \;=A^{{\;}^{\prime \prime }}\cos\left(\pm 2\pi f_{d}t-\emptyset \right) \end{array}$$

(3) $$\begin{array}{l} \text{Q}\left(\text{quadrature}\right)=\text{LPF}\left\{S_{echo}^{*}sin\left(2\pi f_{c}t\right)\right\}\\ \;={-A}^{{\;}^{\prime \prime }}\sin\left(\pm 2\pi f_{d}t-\emptyset \right) \end{array}$$

(4) $$\begin{array}{l} \text{Amp}\left(\text{baseband amplitude}\right)=\sqrt{I^{2}+Q^{2}}=A^{{\;}^{\prime \prime }} \end{array}$$

(5) $$\begin{array}{l} \text{Ph}\left(\text{baseband phase}\right)=\text{Arctan}\left(Q/I\right) \end{array}$$

The Supplementary section provides a detailed explanation of pulse radar signal acquisition and decomposition into in-phase and quadrature components.

### Feature extraction

In the previous work of de Goederen et al. (2021), 38 features were extracted from reconstructed respiratory and motion signals. In that study, the subjects' ages ranged from 2 months to 14 years. They found movement features to be the most important for sleep-wake differentiation, whereas respiration features were most important to distinguish between active and quiet sleep. Preterm infants have more immature cardiorespiratory systems (de Groot et al., 2021) and show other movement patterns during sleep (Bik et al., 2022). This should therefore be taken into consideration during feature extraction.

The current research deals with preterm infants—whose sleep stages are of a more disorganized quality compared to older children (Davis et al., 2004). Therefore, 10 more features (empirical mode decomposition (EMD) and phase features, explained below) were added to help analyze the complexity in the data more thoroughly. A full list of 48 extracted features is summarized in Table 2. In all, 13 features came from the motion signal, 29 features from the respiration rate (RR), and 6 features from the phase of the received signal. A detailed and more technical description of three types of feature extraction is available in the Supplementary section.

**Table 2:** Feature list and description.

**Feature types**	**Category**	**Number**
Average (slow and fast)	Movement	2
Area (slow and fast)	Movement	2
Variance (slow and fast)	Movement	2
Information entropy (slow and fast)	Movement	2
Fast movement ratio	Movement	1
Ratio of EMD Power	Movement	4
Multiscale sample entropy with 1 and 2 units of delay in 10 scales	Breathing	20
Sample and approximate entropy	Breathing	2
Teager Energy in time and frequency domains	Breathing	2
Kat'z fractal dimension	Breathing	1
Variance and average (whole epoch and 30 S centered at each epoch)	Breathing	4
Entropy of binary phase	Phase	6

#### Movement features

There are 52 bins in the baseband data. Each bin was mapped to a specific range of detection. Each 60-S period (equal to observation scoring sampling time) was composed of 1,200 samples. The baseband data can be converted into movement signals by subtracting each bin's signal from consecutive samples to omit detected objects that are static. In this regard, movements in windows of 60 and 1 S were computed as slow and fast movements, respectively. Entropy, average, and standard deviation of amplitude signals were combined into movement features (de Goederen et al., 2021). For further explanation about converting baseband amplitude signal to movement, see Skolnik, 2008. In addition, we added the relative power of 15 dynamical modes from EMD of amplitude signals (Zhao et al., 2016). These groups of EMD features were previously shown to be effective in improving UWB radar's detection algorithms (Diez et al., 2009). In fact, one main advantage of EMD analysis over Fourier decomposition is that EMD has no stationary presupposition of the mechanisms that are generating the signal. So, by these type of features, we could also consider possible nonstationary process of irregular sleep related respiration among preterm infants (Zhao et al., 2016). A more detailed explanation of the EMD algorithm can be found in Flandrin et al., 2003.

#### Respiratory features

Respiratory rate is calculated by counting the rise and fall of chest movements. Therefore, this semi-periodic pattern can be derived by high resolution UWB radar through proper range estimation (He et al., 2020). If the radar can distinguish the range of the chest wall from adjacent moving body parts, the highest peak in the frequency spectrum of the radar amplitude matrix can be detected as the true RR frequency component (Kwasniewska et al., 2019). To do this, the respiratory signal is reconstructed from the baseband amplitude using pulse Doppler processing (Bernfeld, 1967). We employed windows of 60 S (1,200 samples) with an overlap of 95% on amplitude signals. Subsequently, we formed an amplitude matrix of the following dimensions: 1200^*^52 (samples^*^bins).

Using the discrete Fourier transform (DFT) of this matrix, the Doppler frequency at each bin can be found. More specifically, the DFT of each bin is related to the Fourier expansion by periodicity equal to the pulse repetition frequency. As a result, the dominant pattern—which is ideally the RR—corresponds to the maximum frequency of the amplitude matrix. Time, frequency, and energy from this vector were computed as respiratory features, as mentioned in Table 2.

#### Phase features

Phase of received signal is usually ignored in the pulsed-Doppler radar analysis because of the destructive effects of phase noise. However, phase can show the direction of the target's movement, which is a very important Doppler-related characteristic. When considering Equations 2 and 3, one can see that the derivative of the baseband phase signal corresponds to whether the target is approaching or receding. In this regard, it is highly useful to extract from the baseband phase [or tan^−1^($\frac{Q}{I}$)] if the phase derivative is positive or negative. In other words, the positive and negative signs of (unwrapped) phase differentiation can demonstrate whether a moving target is coming toward or going away from the receiver. This information (in accordance with the positive or negative sign of Doppler frequency) may be lost when solely using the amplitude of the received signal. It is important to note that we do not consider the magnitude of the phase, because it is very susceptible to phase noise effects.

To calculate the phase features, we compared phase signs (+ or -) of the five adjacent bins that were most important for respiratory reconstruction, which were operationalized as the bins with the most movements detected. If the phase signs between two bins was different, this was coded as “1.” If the phase signs between two bins were the same, this was coded as “0.” The result was a binary vector that showed the variability in Doppler signs in adjacent bins.

Thus, in each 60-S period, which was equal in length to the interval of sleep scoring, we coded the variability of Doppler signs into binary strings using heuristically found windows of 0.25 S. As the sign of phase differentiation in each bin was compared to the other three, a total of 6 binary vectors were extracted each minute. Each binary vector was considered a phase-coded message. Less repetition of each binary representation (“0” or “1”) in a vector corresponded to higher entropy (Shanmugam, 1979).

**Figure 4:**
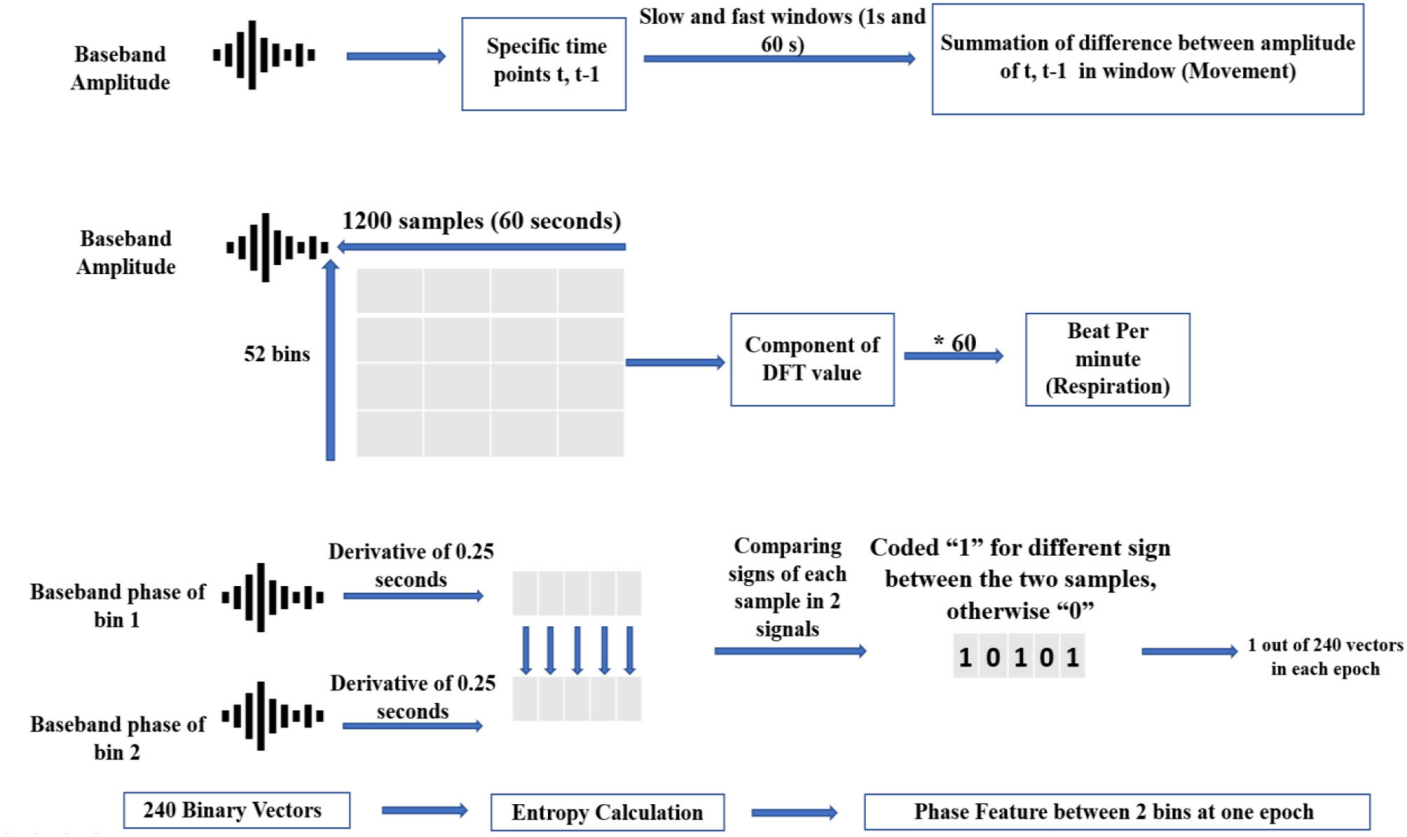
Simple schematic of 3-fold feature extraction from baseband data.

Figure 4 displays the movement, respiratory, and phase feature acquisition from baseband signal through a block diagram, and (Shanmugam, 1979) discuses entropy of binary vectors in detail.

### Classifier, data pooling and evaluation

To classify sleep stages, the following six frequently used machine learning classifiers were compared: 1) support vector machine (SVM), 2) K nearest neighboring (KNN), 3) adaptive boosting (AdaBoost), 4) naive Bayes (NB), 5) decision tree (DTree), and 6) linear discriminant analysis (LDA). For a detailed discussion of each classifier, see Xu (2019). The *Gridsearch* Package in MATLAB (R 2016) was used to find the best learning parameters for each classifier.

Given the unbalanced nature of preterm sleep—with a presumed bias toward AS—a balanced weighted class approach was used to train the classifiers (using empirical prior parameters in MATLAB). To overcome any further biasing effect in the evaluation of our model performance, balanced accuracy and Kappa is also reported.

All classifiers were tested on the performance of QS-AS classification based on 10-fold cross-validation on the pooled data of 10 subjects. The performance of each classifier was evaluated by computing the sensitivity, specificity, and area under the receiver operating curve (AUROC). Furthermore, for each classifier, a Cohen's kappa was calculated with the observations that served as the gold standard. Finally, to show the discriminative power of a single feature toward the output, feature importance was calculated for the best classifier.

## Results

**Figure 5:**
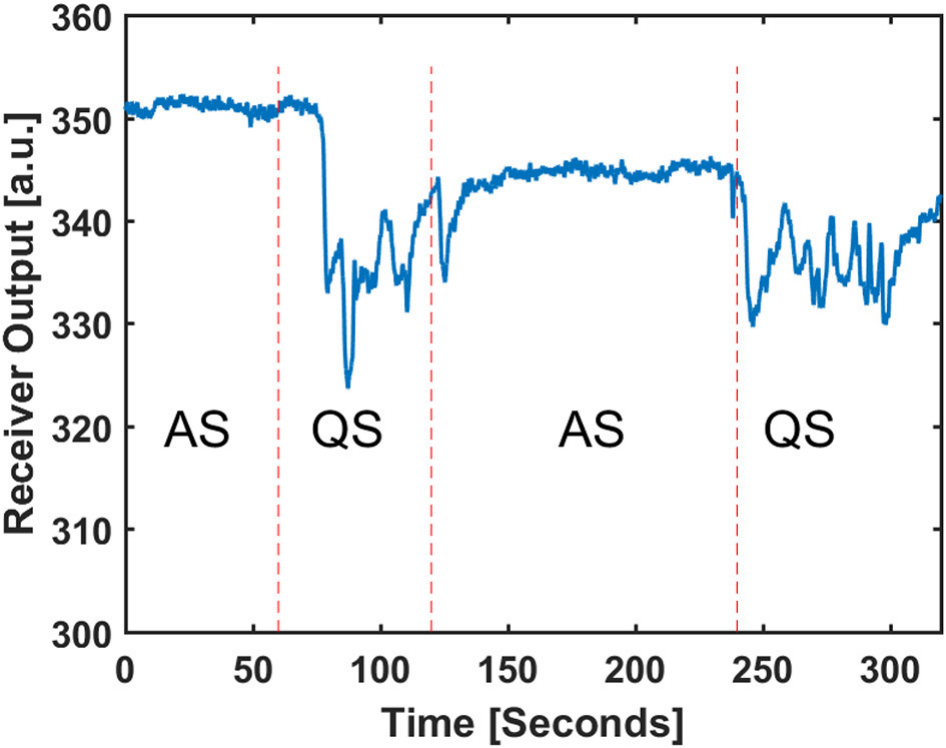
Received baseband amplitude of radar in the target detected range and the related sleep stage scored by video observation.

For a better visualization of raw radar data during the two stages, Figure 5 is plotted to show the discrimination between two AS and QS stages. Figure 5 shows raw radar baseband data for the transition between AS and QS. The displayed data is related to the bin that has the frequency component with the highest power in the frequency spectrum. In an ideal noiseless environment, without frequency modulation of breathing pattern, this bin is capturing the chest wall pattern.

### Patient characteristics

The time of recording was 123 ± 39 min for the 10 subjects. The dataset contained a bias toward AS, as was expected. Therefore, only periods with a high confidence score (>0) were used for AS classification.

Patient characteristics are presented in Table 1. No infants received phototherapy. All infants received caffeine at 8 am as part of routine care. Observation times occurred between 9 am and 7 pm.

### Classification results

The classes were biased in count in favor of AS by a ratio of 2.8:1. After pooling all data, the input matrix consisted of 48 features and 577 tags (60 S observations), which resulted in an input matrix of 48^*^577 that was used to test the classifiers. Table 3 lists the best learning parameters for each classifier.

**Table 3:** Best learning parameters for each classification method obtained by GridSearch.

**Model**	**Best parameters**
SVM	{‘Kernel': Linear};{‘ Box constraints '=0.072}; {‘Solver': SMO};{‘ Kernel scale '=1}; {‘Decision function shape': Ovo}
Naive Bayes	{‘Kernel': Normal – ‘Width'=0.028}
Adaptive Boosting	{‘Number of learning cycles'=479};{'Learning rate'= 0.8}; {‘ Minimum Leaf Size'=4};{‘Max Splits'=58}
Discriminant	{‘Discriminant type ': Linear };{‘Gamma'= 0.07};{‘Delta'=0.002}
K-nearest neighbors	{‘Distance weight': Squared inverse};{‘Number of Neighbors '=12}; {‘NS method: Exhaustive};{‘Break ties ': Smallest}
Decision Tree	{‘Number of nodes'=9 };{‘Split criterion': Deviance};{‘ Minimum Leaf '=14};{‘Max Splits'=7}

**Figure 6:**
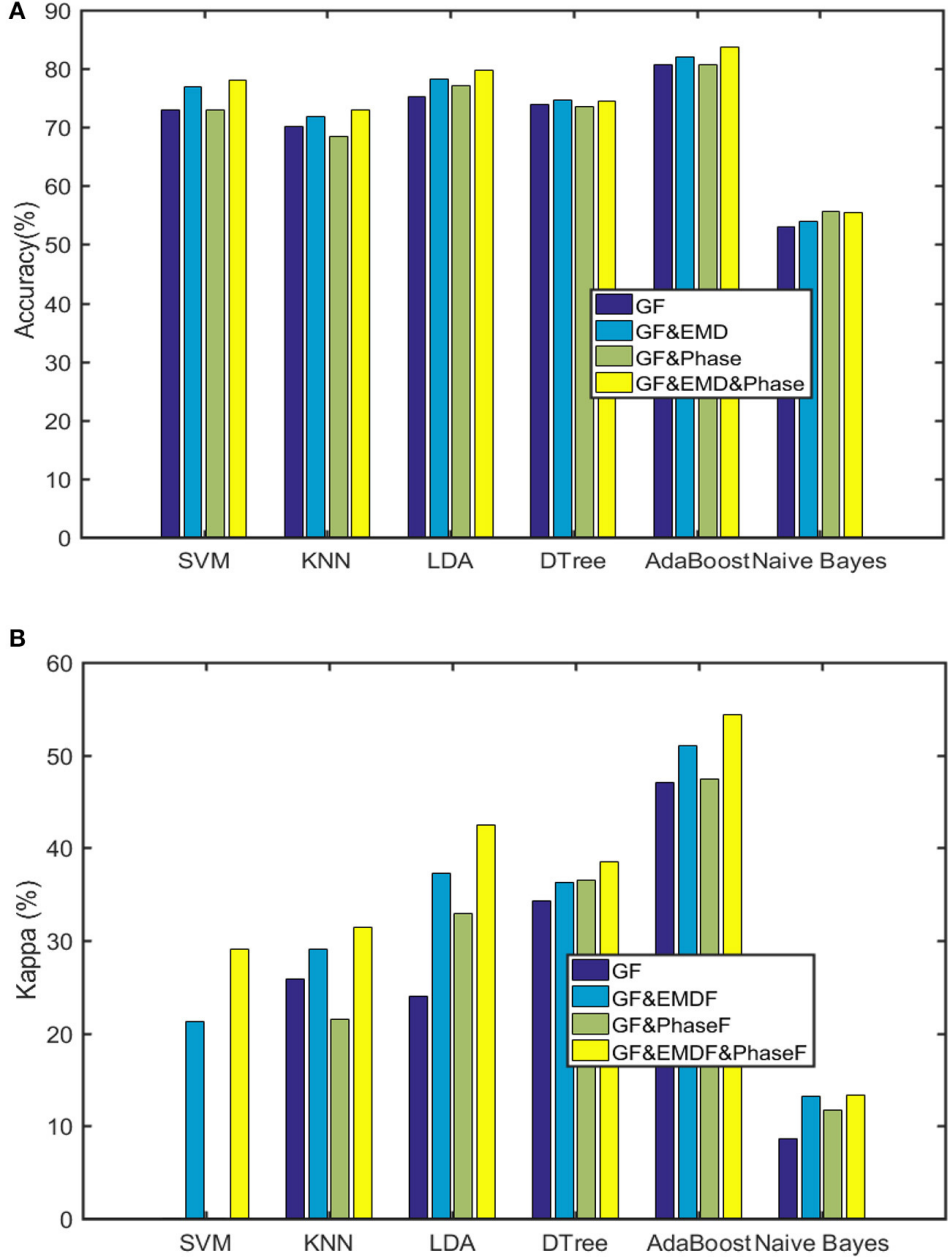
Outcomes of the six machine learning algorithms (GF are the general 38 features utilized in de Goederen et al., 2021). **(A)** Classification accuracy. **(B)** Kappa.

Figure 6 depicts the accuracy and Kappa of the six machine learning approaches on binary classification of sleep stages (AS/QS). The AdaBoost classifier had the best performance (83%). Furthermore, Figure 6A shows the positive effect of the added features on accuracy compared to the original 38 features (GF; de Goederen et al., 2021). Figure 6B shows that the outcomes are still the best for 48 features of AdaBoost for Kappa (which is more robust to imbalanced class distribution than accuracy).

Table 4 provides performance metrics of the best classifier (AdaBoost). Finally, Table 5 lists the most dominant features for the AdaBoost classifier. This confirms that both EMD and phase features possess informative aspects of sleep stages of preterm infants.

**Table 4:** AdaBoost performance on the 48 features as the best result.

**Parameter**	**Value**
F1-Score	0.89
Balanced accuracy	0.81
AUC ROC	0.82
Cohen's Kappa	0.54

**Table 5:** Normalized weight of 5 dominant features.

**Feature**	**Normalized importance weight**
Slow movement average	1
EMD feature of slow movement	0.533
Slow Movement average	0.375
Fast movement area	0.292
Phase entropy (between two bins with most movements)	0.291

### Classification performance

Because the classification is imbalanced, we should be careful about reporting accuracy itself. Imbalanced train datasets are potentially able to lead to high accuracy, while the lower frequent labels are highly misclassified. For example, if an algorithm would classify every sleep stage, as in our sample, it would still reach an accuracy of 64%.

**Figure 7:**
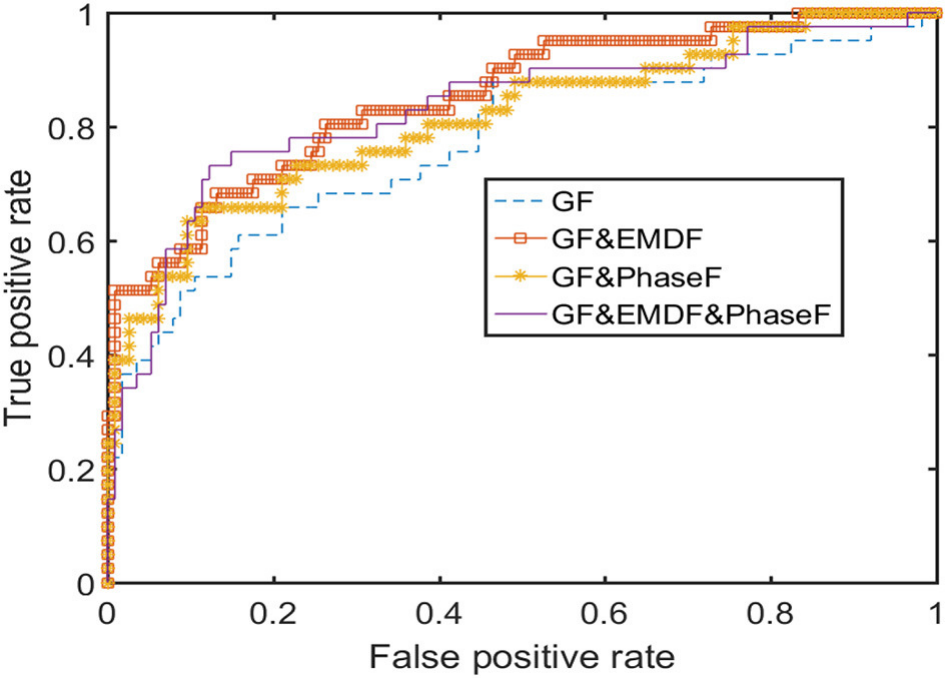
ROC curve for the Adaptive Boosting classification.

To be sure this was not the case in our results, both sensitivity and specificity were incorporated in further outcome measures (e.g., balanced accuracy, which is the average of sensitivity and specificity). The ROC curve for the AdaBoost classifier is displayed in Figure 7. These results show that the classifier worked well, and that misclassification was not inclined toward quiet sleep.

## Discussion

We investigated the feasibility of an UWB radar module to unobtrusively and continuously measure sleep stages in 10 preterm infants between 29 and 34 weeks PMA admitted to the NICU. Six machine learning classifiers were extensively tested. Although preterm sleep data is inherently imbalanced—showing more AS than QS—moderate-to-high accuracy was reached. Using an AdaBoost classifier, AS and QS could be detected in preterm infants, with a performance reaching a Cohen's kappa of 0.54 and a balanced accuracy of 81%.

To train the classifiers, features were extracted from the amplitude and phase of the down-converted radar data. Respiration was reconstructed from the movement signal. This approach resulted in three feature classes: movement, reconstructed respiration, and phase. In our study, the movement features were found to be most important for binary sleep stage differentiation.

The findings were in accordance with de Goederen et al. (2021), also who showed that AdaBoost was the best performing machine learning algorithm for classifying sleep stages in children using UWB radar data. While they showed movement features to be more important than respiratory features in sleep-wake stage classification, the respiratory features were most important to differentiate between sleep stages. In contrast, the current study identified movement as the most important feature for sleep stage classification, as opposed to respiratory-based features.

A possible explanation for this discrepancy is the immature cardiorespiratory systems of preterm infants (de Groot et al., 2021). Furthermore, in infants, AS is characterized by a high level of motor activity, whereas QS is characterized by motor quiescence (Bik et al., 2022), while children have already developed muscle paralysis during REM sleep (Anders et al., 1995; Ferber, 1995). Finally, chest wall movements are subtle in preterm infants and can hardly dominate the baseband amplitude matrix pattern. More specifically, these small movements have only a small effect on the overall signal, even when they are extracted separately. Nevertheless, respiratory features were shown to have a clear complementary role in predicting sleep stages for our cohort, as performance improved when respiratory features were used in the classifier.

One new insight from this study is the importance and benefit of incorporating both the phase of received signal (through phase features) and it's nonstationary nature (by EMD). As shown in Table 5, EMD makes the 2nd and phase the 5th most important feature in sleep classification. So, the amount of irregularity in respiration of preterm infants needs to also be scrutinized both by detection of directionality of chest movement (by phase feature) and its nonstationary patterns (by EMD features).

The current study reached a Cohen's kappa of 0.54 and balanced accuracy of 81%. Although de Goederen et al. (2021) did obtain a higher Cohen's kappa when distinguishing sleep from wake, this was not the case when distinguishing between REM, non-REM, and wake. Research by Lee et al. (2021) used UWB radar to classify sleep/wake states in 4 (near) term infants. In these older infants, they reached a slightly lower overall performance compared to our research, with a Cohen's kappa of 0.49 and an overall accuracy of 75.2%.

Similar to our study, Lee et al. (2021) found movement to be the most important feature in this algorithm. However, they identified more movement during sleep (e.g., a muscle twitch or startle during AS) as the cause of discrepancies, for example, when the stage was identified as sleep by the gold standard and as wake by the radar. It is important to note that Lee et al. distinguished between sleep and wake, while the current study only distinguished between AS and QS.

## Limitations

The UWB radar picks up on any moving objects within range. This can be a limitation, as people's movement around the device might cause unwanted signals. In our cohort, we did not perform any radar measurements during clinical care.

Secondly, the classifiers that we compared in this study were all trained to differentiate between AS and QS. The choice to not include waking activity was based on low availability of periods spent awake (1 out of 400 1-min epochs on average for 10 cases). However, when preterm infants are awake, behavior is relatively similar to AS (Bik et al., 2022). Despite this limitation, a QS/non-QS distinction allows researchers to distinguish a sleep state trend, which can be used as a proxy marker of brain development and to steer future care (Moghadam et al., 2022).

Finally, the current algorithm is only useable in a specific age range (29–34 weeks PMA). However, preterm infants older than 28 weeks PMA are thought to benefit the most from the advantages of continuous bedside sleep stage classification. Before 28 weeks of PMA, sleep stages have often not been clearly consolidated yet (Mirmiran et al., 2003; Dereymaeker et al., 2017; Bourel-Ponchel et al., 2021; Georgoulas et al., 2021) and between 29 and 34 weeks PMA, most of the NICU care is elective and can be adjusted to behavioral needs.

## Future perspectives

In the current research, four EMD and six phase features were added to the selection used by de Goederen et al. (2021). Future research could investigate the useability of other features to improve classification in infants and children. The added value of EMD suggests that dynamic variations of the radar may deserve some investigation. Furthermore, the added value of phase features suggests that the directionality of the Doppler and breathing dynamics may be a starting point for future research.

Moreover, in this study, we have not applied any specific feature selection method on our data. The reason for this is the different outcomes of various feature selection methods. For this study, the main goal was to show how feature extraction from UWB radar can be helpful in sleep stage prediction among preterm infants. Also, methods like AdaBoost are more robust to feature selection changes, so we used machine learning algorithms to find the dominant features. One interesting idea for future works on this study is to see how different feature selection methods affect classification outcomes.

Additionally, we can use our algorithm on a larger sample size to see how it works for leave-subject-out cross-validation. In this way, we can tune our model to be more subject-independent and generalized. There is also the option of adding infants with more wake states to check the feasibility of our features in multi-class analysis.

In our study, unlike to de Goederen et al. (2021), the movement features were found to be most important for binary sleep stage differentiation. This shows that respiratory features for preterm infants are not completely illustrating their sleep stages. An important factor that should not be overlooked is the occurrence of periodic breathing among preterm infants, which can cause movements due to fast and shallow breaths (Dereymaeker et al., 2017). Although this pattern of respiratory-related motion typically lasts no more than 10 S (Mirmiran et al., 2003), which is shorter than the duration of each of our labeled tags, it can produce frequency harmonics larger than the peak frequency considered as a respiratory periodic pattern. However, the duration of periodic breathing is shorter than 18% of the duration of slow-movement related features, including the three most dominant movement features (slow movement average and variance, and EMD of slow movement). Therefore, it is unlikely that periodic breathing will significantly affect these features.

An interesting topic for future research will be to explore the sensitivity of shorter tag durations (less than the 60-S epochs that we chose) of sleep classification to the higher frequency harmonics of periodic breathing. More specifically, it may be interesting to assess the possible trade-off between the minimum resolution time of possible sleep stage classification and the dominance of the monochromatic frequency pattern of respiratory rate for sleep stage classification. One may expect that the time resolution (time length of each sleep stage tag) of smaller values might need a multi-tone analysis of respiratory rate extraction to prevent a disproportionate influence of periodic breathing on sleep classification. However, the boundaries and thresholds for epoch length should be a topic of future investigations.

An important aspect of neonatal sleep stage classification is defining the “gold standard.” In this paper, the manual BeSSPI system has been applied for scoring sleep because it is the first validated behavioral scoring system for preterm infants. It must be noted that usually, the computer-based automated sleep scoring systems lead to a broader range of Cohen's kappa scores (0.6 to 0.95) than human scoring systems for machine learning classification algorithms (Penzel and Conradt, 2000; Louis et al., 2004; Hamida and Ahmed, 2013). Among the human based manual sleep classification systems for preterm infants, Cohen's kappas range between 0.31 (Brazelton) and 0.96 (Prechtl) (Bik et al., 2022). Future work could explore how our algorithm performs when based on other sleep assessment methods that could be used in preterm infants–such as EEG (Ansari et al., 2018).

The AdaBoost classifier is highly viable to noise bias in the data (Cao et al., 2012). However, when comparing the AdaBoost results to the other classifiers, the AdaBoost results excel on every index. This demonstrates that noise did not have a major disruptive effect on the data and sleep prediction, possibly because all subjects were relatively stable and undisturbed during recording. Therefore, any available noise is probably due to motion artifacts–which provide information about the behavioral state of the infant. Nevertheless, it will be interesting to evaluate the sensitivity of sleep classification by radar to the peripheral noise in the NICU (e.g., due to people walking by or opening the incubator) in future research.

Finally, we believe that future research should look into combining different unobtrusive monitoring data to optimize classification accuracy. It is worth investigating if adding data from video, heart rate, and other modalities to radar signals can improve sleep assessment of preterm infants.

## Conclusions

The current research showed that it seems feasible to distinguish active from quiet sleep stages in preterm infants in a clinical neonatal intensive care setting using UWB radar data. Classification using radar data can reach a high balanced accuracy, despite the inherent imbalance in the data. Significant features from respiratory and movement signals improved sleep stage classification accuracy compared to a previously published algorithm. Considering further potential improvements that can be made, UWB radar is a very promising tool for continuous non-obtrusive sleep stage detection in preterm infants in the neonatal intensive care unit.

## Data availability statement

The datasets presented in this article are not readily available because this is a hospital data and cannot be accessed by people outside WKZ hopital. Requests to access the datasets should be directed to emad.arasteh@gmail.com.

## Ethics statement

The studies involving human participants were reviewed and approved by permission to use pseudonymized patient data was obtained from parents using the consent form and from the local Medical Ethical Review Committee (METC number 21-816/C). Written informed consent to participate in this study was provided by the participants' legal guardian/next of kin.

## Author contributions

EA: method analysis, data analysis, coding, visualization, and writing and editing manuscript. EG: method analysis, data recording, sleep scoring, visualization, and writing and editing manuscript. DE: method analysis, data recording, and writing and editing manuscript. TA, RG, XL, and MB: method analysis and writing and editing manuscript. JD: method analysis, supervision, and writing and editing manuscript. All authors contributed to the article and approved the submitted version.
