# Supplementary material for: Unobtrusive cot side sleep stage classification in preterm infants using ultra-wideband radar
Source: Front Sleep. 2023 Jun 15;2:1150962. doi: 10.3389/frsle.2023.1150962 (PMC12713870; doi:10.3389/frsle.2023.1150962)
Supplement: Supplementary file 1 [file Data_Sheet_1.docx]

1. **Radar data ​​processing**

If the carrier frequency is f_c_, the reference transmitted signal can be assumed to be a continuous wave sinusoidal signal – which is modulated by pulse signal – with amplitude A:

$S_{ref}=A Sin(2\pi f_{c}t)\pi(\frac{t}{\tau})$; 0<t<T (1)

T is the pulse repetition and $\tau$ is the pulse width. $\pi(\frac{t}{\tau})$ defines the duration of pulse with a nonzero value ($\tau$ out of each T seconds). Then the received echo influenced by the Doppler frequency of the moving target ($f_{d}$) can be written as a signal with lower amplitude $A^{'}$ and a phase shift $\emptyset$ dependent on the distance of target and carrier frequency (just for the pulse width duration):

$S_{echo}=A^{'} Sin(2\pi\left( f_{c}\pm f_{d} \right)t-\emptyset)$ (2)

The $\pm$ symbol is used to distinguish between the approaching or receding target. In the case of baby breathing, different sign can be interpreted as inhale and exhale patterns. So, a proper analysis of phase is potentially able of distinguishing patterns of inhale and exhale. As a result, we aim to incorporate phase in our algorithm to see how it can help us to achieve better informative features. Keep in mind that, some moments of inhale and exhale can have the same reflected amplitude (or at least there is no reason they can’t), but are different in the sign of phase. This is sth of importance that may be helpful (-which we will show in the result section that will be helpful-) in better sleep stage classification.

A heterodyne receiver synced with a transmitter can utilize a mixer to down-convert the signal into the baseband domain (LPF stands for low pass filter). Baseband is the frequency band that message information (here the Doppler frequency and speed) can be extracted without (usually considerable) carrier influence. The baseband signal is decomposed into in-phase and quadrature components. It incorporates in-phase and quadrature signals to compute orthogonal elements of baseband signal:

I (in-phase)= LPF{$S_{echo}*cos(2\pi f_{c}t$)}= $A^{''} cos(\pm2\pi f_{d}t-\emptyset)$ (3)

Q (quadrature)= LPF{$S_{echo}*sin(2\pi f_{c}t$)}= ${-A}^{''} sin\left( \pm2\pi f_{d}t-\emptyset\right)$ (4)

Amp (baseband amplitude)=$\sqrt{I^{2}+Q^{2}}$=$A^{''}$ (5)

Ph (baseband phase)= Arctan($Q/I$) (6)

1. **Feature Extraction**

**1) Movement features:** There are 52 bins in the baseband data. Each bin is mapped to a specific range of detection. Each 60-second epoch (equal to observation scoring sampling time) is composed of 1200 samples. The baseband data can be converted into movement signals by subtracting from consecutive samples of each bin to omit static detected objects. In this regard, movements in windows of 60 and 1 seconds are computed as slow and fast movements, respectively.

We also added relative power of 15 number of dynamical modes from empirical mode decomposition (EMD) of amplitude signals. In consecutive steps, EMD extracts different multi-tone harmonics from the original signal. These harmonics are called intrinsic mode functions (IMF) that have nearly zero average minimum and maximum extremum envelopes (through a process called sifting algorithm). In some researches, these type of EMD features have shown to improve radar processing performance. EMD is applied on 4 set of signals from movements: movements computed in windows of 1, 10, 30, and 60 seconds.

**2) Respiratory features:** First, we reconstruct the respiratory signal from baseband amplitude. This is done by pulse Doppler processing. We employ windows of 60 seconds (1200 samples) with an overlap of 95 % on amplitude signals. Then we form an amplitude matrix of dimension 1200*52 (samples*bins).

The amplitude matrix is the baseband amplitude distribution among each bin through every scoring epoch of 60 seconds. As the main change in (the optimal condition of) our experience is the chest wall movement of infant, we try to extract this movement as respiratory pattern. Discrete Fourier transform (DFT) of this matrix aids to find the Doppler frequency at each bin. This is because DFT of each bin is related to Fourier expansion by periodicity equal to pulse repetition frequency. As a result, the dominant pattern which is ideally the respiration rate corresponds to the maximum frequency of the amplitude matrix. To be more specific, supposing suitable clutter (or noise) rejection and negligible false alarms, the frequency with highest poser spectral share, is related to Doppler caused by respiration. As a result, these extracted frequencies (multiplied by 60 to represent beats per minute (BPM)) are put consecutively in a vector to represent reconstructed respiration. Time, frequency, and energy from this vector are computed as respiratory features mentioned in Table 3.

**3) Phase features:** Phase of received signal is usually ignored in the pulsed-Doppler radar analysis because of destructive effects due to phase noise. However, phase can show one very important Doppler related information. Looking into equation 3 and 4, it is obvious that derivative of baseband phase signal corresponds to whether the target is approaching or receding. In this regard, one informative aspect of base-band phase (or tan^-1^($\frac{Q}{I}$)), is the sign of phase derivative. First, the phase signal should be unwrapped to add up for instantaneous jumps. The positive and negative signs of phase differentiation can demonstrate whether a moving target is coming toward or going away from the receiver. This information (in accordance with the sign of Doppler frequency) may be lost in the amplitude of the received signal. There is no reason to receive different amplitudes when chest ball movements are at a backward or inward position in a multiple scattered environment like our test set-up. As a result, we seek additive and complementary information among phase signals besides amplitude.

We do not consider the magnitude of the phase, because it is very susceptible to phase noise effects.
We compare phase signs of the 4 most important adjacent bins (with the most movements detected). Different phase sign between two bins is coded as “1”, and the same signs are coded as “0”. In this manner, the binary resultant vector can show how much different is Doppler signs in adjacent bins. In each 60-second epochs (equal to the interval of sleep scoring), we code this aspect of phase into binary strings among windows of 0.25 seconds (found heuristically). We compute these binary phase codes in the 4 most important bins for respiratory reconstructions (the 4 most frequent bins corresponding to maximum movement). Sign of phase differentiation in each bin is compared to the other 3. So, for 4 bins, we extract 6 binary vectors of 1 and 0.

Each of these binary vectors in 60 second epochs is considered a phase-coded message. The less repeated each binary representation of a vector in epoch, the higher its information (entropy) . In the worst case, if all the vectors in an epoch are the same binary representations, then entropy in that epoch is zero.

So, the entropy feature (H) for each epoch is calculated based on the probability of occurrence of each binary 10-bit message in the epoch:

H=$-\sum_{i=1}^{N} l{og}_{2}(p_{i}){.p}_{i}$; $p_{i}=\frac{Number of vectors having i-th bit representation}{60}$ (7)

N is number of unique binary vector in 240 vectors of each epoch. Finally, we will extract 6 features out of the baseband phase values.

Figure 3.b. displays the movement, respiratory, and phase feature acquisition from baseband signal through a block diagram.

1. **MATLAB Package**

MATLAB classification packages enable us to define prior classification categorical ratio. It allows researchers to set ‘prior’ as ‘empirical’ in “fitting” the model and it magnifies the importance of the smaller (in quantity) class in importance of weight calculation. So, if a weight of a feature is more deterministic of the smaller class it will be magnified by the ratio number of bigger class samples/number of smaller class samples). In addition, through the “Stratified 10-fold” cross validation procedure, we can make sure that any fold has the same ratio between the two AS and QS classes for training. In this manner, ratio of imbalance is kept through whole classification process

1. **Subject Cross Validation**

To check the effect of subject cross validation on the performance of AdaBoost model, we split the data into 60% (6 subjects) for training and 40% (4 subjects) for the test. The subjects were chosen so the full datasets had the least possible class imbalance difference from the total dataset’s categorical bias. To minimize bias in our data, the test subjects were split in 5 groups as follows:

G1= {1 3 7 8}; G2= {1 2 3 5}; G3={4 7 9 10}; G4={4 5 6 10};G5={2 5 8 9};

Within this division, eight subjects have been added twice, one subject is added once and one subject is added three times. The accuracy and balanced accuracy are depicted in Fig S2. Each test group shows an accuracy of approximately 70%. This is a little lower than the pooled dataset but still reliable.

Figure S1. The accuracy and balanced accuracy for the five G1 to G5 test groups.

1. **Confusion Matrixes**

“The Balanced accuracy, Cohen’s kappa, F1-Score, and AUC-ROC are all computed from confusion matrix. Here we report the matrix itself:

| Model | Confusion Matrix  [True QS False AS  False QS True AS] |
| --- | --- |
| SVM | 39 116  10 410 |
| KNN | 78 77  79 341 |
| LDA | 70 85  31 389 |
| DTree | 95 60  87 333 |
| AdaBoost | 89 66  28 392 |
| Naive Bayes | 102 53  203 217 |
